# Supplementary material for: The Synergistic Anti-Tumor Activity of EZH2 Inhibitor SHR2554 and HDAC Inhibitor Chidamide through ORC1 Reduction of DNA Replication Process in Diffuse Large B Cell Lymphoma
Source: Cancers (Basel). 2021 Aug 24;13(17):4249. doi: 10.3390/cancers13174249 (PMC8428225; doi:10.3390/cancers13174249)
Supplement: Supplementary file 1 [file cancers-13-04249-s001.zip › cancers-1239699-supplementary/cancers-1239699-supplementary.pdf]

cancers

MDPI

# Supplementary Materials: The Synergistic Anti-Tumor Activity of EZH2 Inhibitor SHR2554 and HDAC Inhibitor Chidamide through ORC1 Reduction of DNA Replication Process in Diffuse Large B Cell Lymphoma

Xing Wang, Dedao Wang, Ning Ding, Lan Mi, Hui Yu, Meng Wu, Feier Feng, Luni Hu, Yime Zhang, Chao Zhong, Yingying Ye, Jiao Li, Wei Fang, Yunfei Shi, Lijuan Deng, Zhitao Ying, Yuqin Song and Jun Zhu

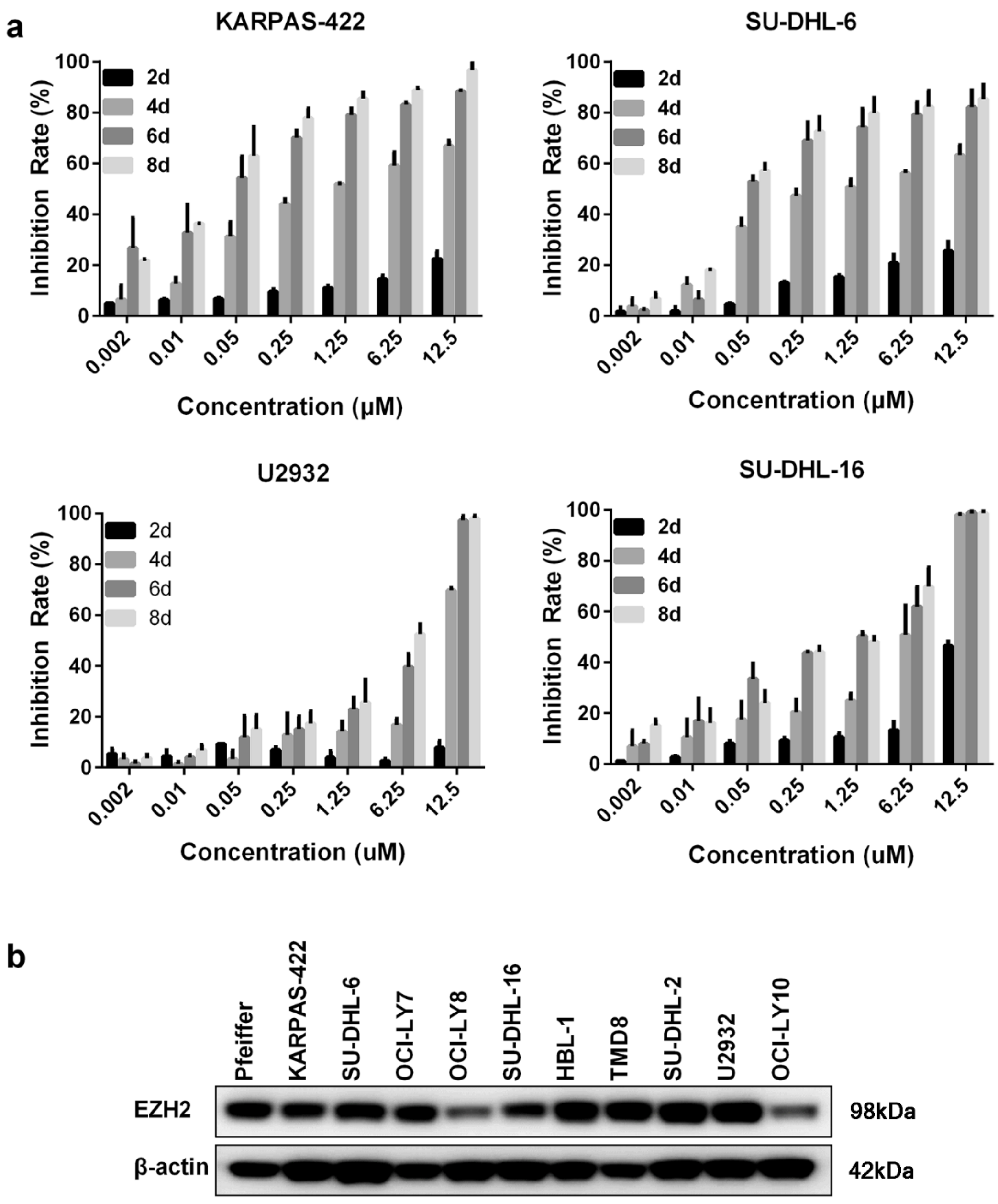


**Figure S1.** SHR2554 inhibited proliferation in a time- and dose-dependent manner in DLBCL cell lines. (**a**) Four DLBCL cell lines were treated with SHR2554 for indicated days and cell viability was measured by Cell Titer-Glo luminescent cell viability assay. Inhibition rates were calculated by (1 − dosing/vehicle) × 100%. (**b**) EZH2 expression was detected by western blot in DLBCL cell lines.

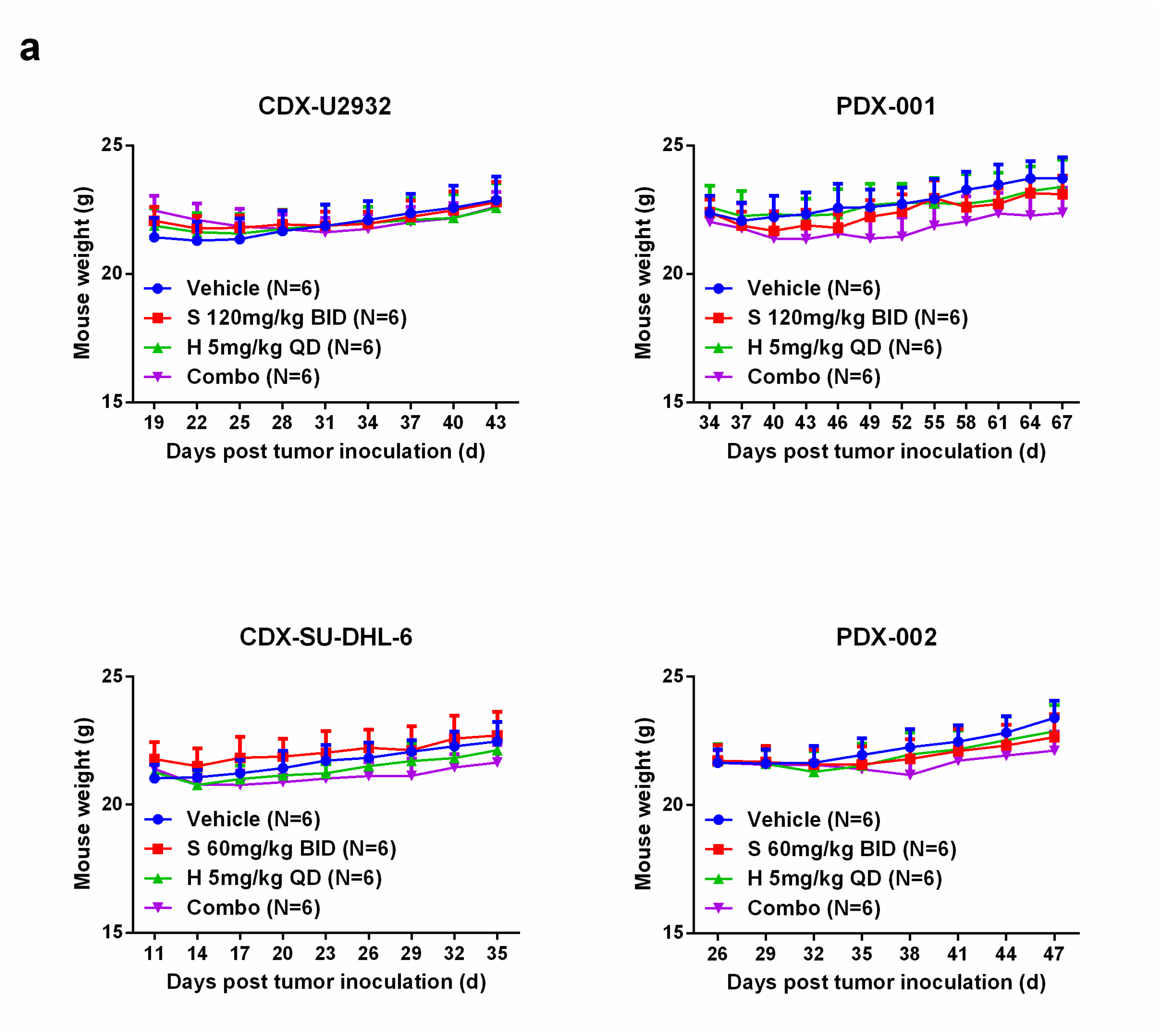


**Figure S2.** Combination of SHR2554 and HBI8000 exhibited synergistic antitumor effect in DLBCL models in vivo. (**a**). Mouse body weight curves derived from four models. Basic clinicopathological features of these DLBCL samples contributed to PDX models are as follows: 1.PDX001-EZH2 WT: non-GCB, CD20 (+), CD3 (-), CD5 (-), Bcl-2 (+), Bcl-6 (+), CD10 (-), CMYC (30%+), CYCLIND1 (-), Ki-67 (80%+), MUM-1 (+) 2.PDX002-EZH2 Y641N: GCB, CD20 (+), CD3 (-), CD5 (-), Bcl-2 (+), Bcl-6 (+), CD10 (+), CMYC (80%+), CYCLIND1 (-), Ki67 (90%+), MUM-1 (-).

## Figure 1 c and e

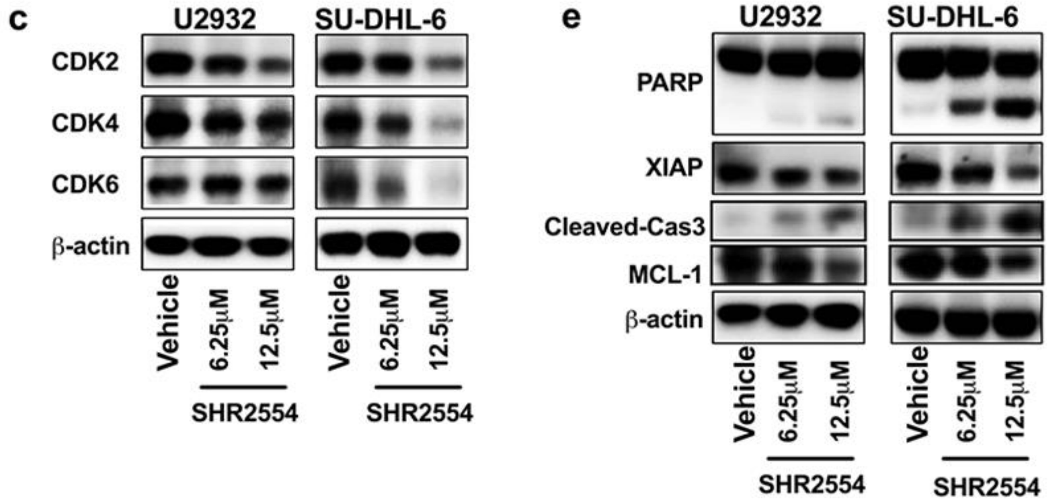


| Figure1c | U2932 | | | DHL-6 | | |
|---|---|---|---|---|---|---|
| CDK2 | 1 | 0.725 | 0.362 | 1 | 0.860 | 0.457 |
| CDK4 | 1 | 0.772 | 0.624 | 1 | 0.672 | 0.224 |
| CDK6 | 1 | 1.07 | 0.802 | 1 | 0.593 | 0.213 |
| β-actin | 1 | 1.22 | 0.97 | 1 | 1.13 | 1.07 |

| Figure1e | U2932 | | | DHL-6 | | |
|---|---|---|---|---|---|---|
| PARP | 1<br>1 | 1.05<br>2.27 | 1.17<br>3.94 | 1<br>1 | 0.94<br>3.59 | 0.80<br>5.59 |
| XIAP | 1 | 0.76 | 0.62 | 1 | 0.80 | 0.48 |
| C-Cas3 | 1 | 1.89 | 2.64 | 1 | 1.49 | 1.71 |
| MCL-1 | 1 | 0.96 | 0.58 | 1 | 0.84 | 0.67 |
| β-actin | 1 | 1.11 | 1.21 | 1 | 1.07 | 1.03 |

## Figure 3b

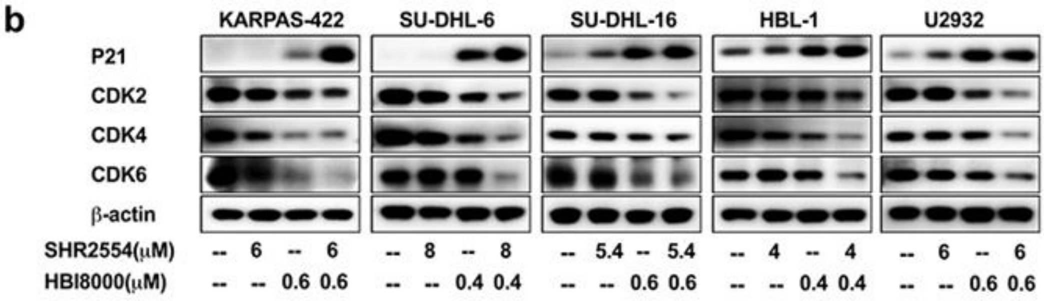


| Figure 3b | KARPAS-422 | | | | DHL-6 | | | | DHL-16 | | | | HBL-1 | | | | U2932 | | | |
|---|---|---|---|---|---|---|---|---|---|---|---|---|---|---|---|---|---|---|---|---|
| P21 | 1 | 0.88 | 2.02 | 4.29 | 1 | 1.02 | 3.47 | 3.68 | 1 | 1.68 | 3.19 | 3.26 | 1 | 1.09 | 1.53 | 1.84 | 1 | 1.84 | 3.62 | 3.28 |
| CDK2 | 1 | 0.74 | 0.51 | 0.40 | 1 | 0.69 | 0.46 | 0.26 | 1 | 0.94 | 0.58 | 0.31 | 1 | 0.85 | 0.76 | 0.6 | 1 | 0.94 | 0.64 | 0.37 |
| CDK4 | 1 | 0.54 | 0.25 | 0.23 | 1 | 0.76 | 0.58 | 0.33 | 1 | 0.97 | 0.79 | 0.66 | 1 | 0.69 | 0.48 | 0.34 | 1 | 0.97 | 0.93 | 0.45 |
| CDK6 | 1 | 1.09 | 0.60 | 0.32 | 1 | 1.12 | 1.04 | 0.45 | 1 | 0.96 | 0.68 | 0.53 | 1 | 1.19 | 1.02 | 0.59 | 1 | 0.72 | 0.61 | 0.41 |
| β-actin | 1 | 1.13 | 1.02 | 0.94 | 1 | 0.93 | 0.99 | 0.91 | 1 | 1.01 | 0.87 | 0.99 | 1 | 0.90 | 0.99 | 0.97 | 1 | 0.98 | 1.19 | 1.04 |

# Figure 3d

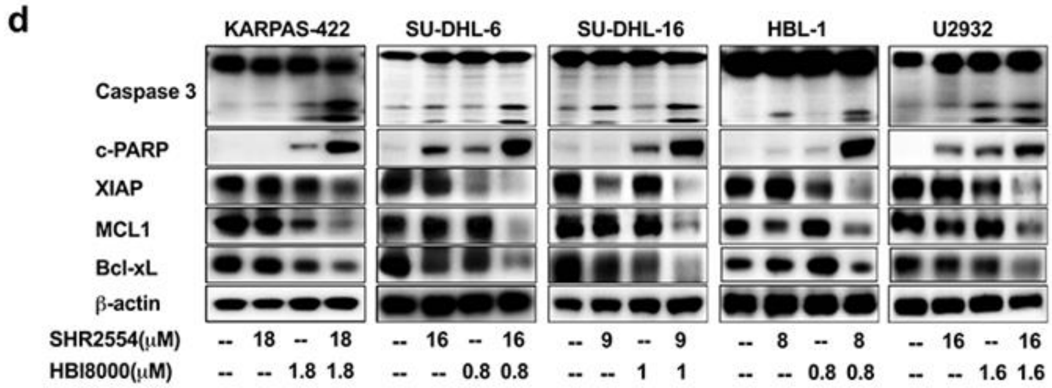


| Figure 3d | KARPAS-422 | | | | DHL-6 | | | | DHL-16 | | | | HBL-1 | | | | U2932 | | | |
|---|---|---|---|---|---|---|---|---|---|---|---|---|---|---|---|---|---|---|---|---|
| Caspase3 | 1 | 0.91 | 4.27 | 11.5 | 1 | 1.65 | 1.49 | 2.24 | 1 | 1.29 | 1.02 | 1.41 | 1 | 2.19 | 1.92 | 4.32 | 1 | 1.30 | 1.38 | 1.47 |
| c-PARP | 1 | 1 | 19.5 | 65.9 | 1 | 4.89 | 3.96 | 9.33 | 1 | 0.68 | 2.49 | 5.54 | 1 | 1.17 | 1.55 | 8.50 | 1 | 15.69 | 19.58 | 28.66 |
| XIAP | 1 | 1.03 | 0.98 | 0.72 | 1 | 0.97 | 0.61 | 0.28 | 1 | 0.50 | 0.89 | 0.23 | 1 | 1.21 | 0.85 | 0.57 | 1 | 1.05 | 0.74 | 0.37 |
| MCL1 | 1 | 0.86 | 0.52 | 0.36 | 1 | 1.16 | 1.22 | 0.30 | 1 | 0.96 | 0.97 | 0.40 | 1 | 0.78 | 1.23 | 0.62 | 1 | 0.81 | 0.91 | 0.48 |
| Bcl-XL | 1 | 0.96 | 0.69 | 0.46 | 1 | 0.88 | 0.84 | 0.31 | 1 | 0.83 | 0.72 | 0.43 | 1 | 1.23 | 1.63 | 0.75 | 1 | 0.99 | 1.03 | 0.76 |
| β-actin | 1 | 0.90 | 1.12 | 1.13 | 1 | 1.17 | 1.09 | 0.98 | 1 | 1.20 | 1.37 | 1.54 | 1 | 1.12 | 1.02 | 0.90 | 1 | 0.93 | 0.99 | 0.82 |

# Figure 3e

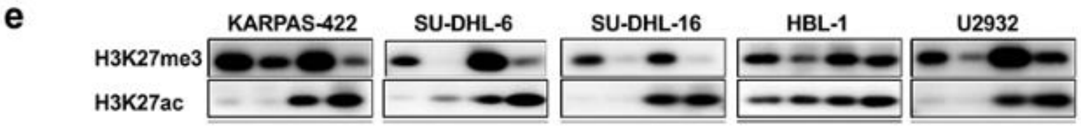


| Figure 3e | KARPAS-422 | | | | DHL-6 | | | | DHL-16 | | | | HBL-1 | | | | U2932 | | | |
|---|---|---|---|---|---|---|---|---|---|---|---|---|---|---|---|---|---|---|---|---|
| H3K27me3 | 1 | 0.62 | 1.1 | 0.32 | 1 | 0.03 | 2.85 | 0.60 | 1 | 0.29 | 1.04 | 0.28 | 1 | 0.8 | 1.21 | 1.05 | 1 | 0.55 | 1.69 | 0.97 |
| H3K27ac | 1 | 1.15 | 3.25 | 4.68 | 1 | 2.54 | 4.77 | 7.92 | 1 | 1.18 | 6.19 | 7.1 | 1 | 1.18 | 1.38 | 1.77 | 1 | 0.67 | 4.99 | 6.92 |

# Figure 5d

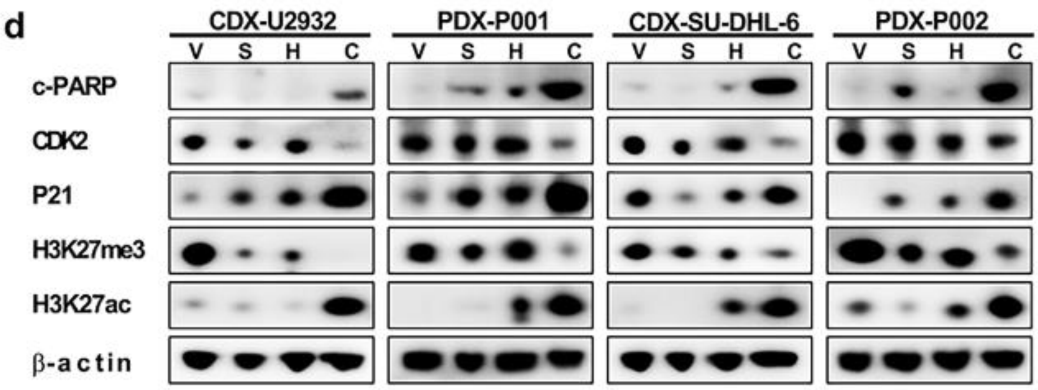


| Figure 5d | CDX-U29832 | | | | PDX-P001 | | | | CDX-SU-DHL-6 | | | | PDX-P002 | | | |
|---|---|---|---|---|---|---|---|---|---|---|---|---|---|---|---|---|
| | V | S | H | C | V | S | H | C | V | S | H | C | V | S | H | C |
| c-PARP | 1 | 1.10 | 1.08 | 2.25 | 1 | 2.08 | 1.95 | 4.19 | 1 | 0.95 | 1.27 | 4.20 | 1 | 1.97 | 1.36 | 4.19 |
| CDK2 | 1 | 0.83 | 1.32 | 0.8 | 1 | 0.82 | 1.01 | 0.40 | 1 | 0.79 | 1.46 | 0.72 | 1 | 0.99 | 0.77 | 0.60 |
| P21 | 1 | 2.12 | 2.36 | 4.54 | 1 | 1.81 | 1.90 | 3.25 | 1 | 0.53 | 0.77 | 1.38 | 1 | 3.96 | 4.26 | 7.96 |
| H3K27me3 | 1 | 0.3 | 0.43 | 0.18 | 1 | 0.72 | 1.13 | 0.34 | 1 | 0.74 | 0.50 | 0.49 | 1 | 0.51 | 0.63 | 0.35 |

| | | | | | | | | | | | | | | | | |
|---|---|---|---|---|---|---|---|---|---|---|---|---|---|---|---|---|
| H3K27ac | 1 | 1.03 | 0.76 | 4.04 | 1 | 1.24 | 4.03 | 5.91 | 1 | 0.88 | 2.67 | 4.32 | 1 | 0.49 | 1.24 | 2.42 |
| β-actin | 1 | 0.86 | 1.01 | 1.11 | 1 | 0.78 | 1.04 | 0.80 | 1 | 0.87 | 1.01 | 1.26 | 1 | 1.18 | 1.15 | 1.17 |

## Figure S1b

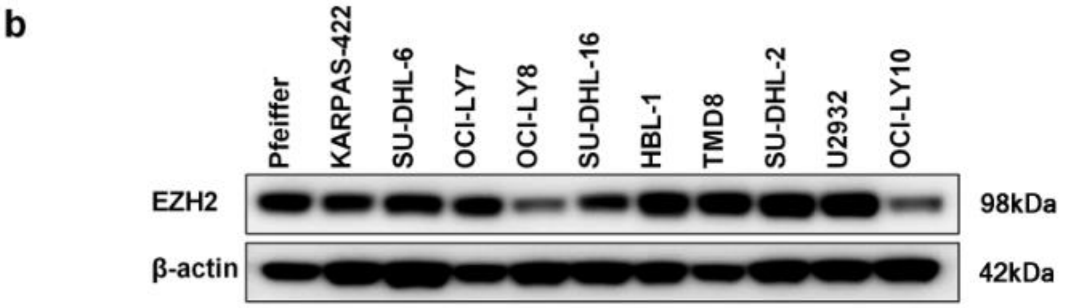


| Figure S1b | pfeiffer | KARPAS 422 | SU-DHL6 | OCI-LY7 | OCI-LY8 | SU-DHL16 | HBL-1 | TMD8 | SU-DHL2 | U2932 | OCI-LY10 |
|---|---|---|---|---|---|---|---|---|---|---|---|
| EZH2 | 1 | 0.92 | 1.15 | 0.97 | 0.65 | 0.85 | 1.18 | 1.16 | 1.26 | 1.30 | 0.65 |
| β-actin | 1 | 1.18 | 1.55 | 0.96 | 1.20 | 1.28 | 1.01 | 0.89 | 1.18 | 1.22 | 1.32 |
